# Specific immunosuppression by mixed chimerism with bone marrow transplantation after *Staphylococcal* Enterotoxin B pretreatment could prolong corneal allograft survival in mice

**Published:** 2012-04-18

**Authors:** Yingnan Zhang, Zhiqiang Pan, Yu Chen, Ying Jie, Yan He

**Affiliations:** 1 Beijing Tongren Eye Center, Beijing Tongren Hospital, Capital Medical University, Beijing Ophthalmology & Visual Science Key Lab, Beijing, China; 2 Department of Immunology, General Hospital of PLA, Beijing, China

## Abstract

**Purpose:**

We assessed the combined use of *Staphylococcal* Enterotoxin B (SEB) superantigen pre-treatment along with allogeneic bone marrow transplant (BMT) to induce immune suppression condition and inhibit corneal keratoplasty rejection in mice.

**Methods:**

BALB/C (H-2d) mice were both BMT and corneal allografts donors and C57BL/6(H-2b) mice were recipients. Prior to BMT, recipients received single injections of either SEB, cyclophosphamide (CYP), or normal saline (NS). Allogenic corneal penetrating keratoplasty was performed 7 days after BMT. Bone marrow chimerisms in recipients (donor major histocompatibility complex-II H2-d) were determined on Days 14, 28, and 56 post-BMT. Recipient immune response was assessed by mixed lymphocyte reactions (MLR) using splenocytes from C57BL/6 mice as responders in co-culture with stimulator cells from C57BL/6 (isogeneic), BALB/C (allogeneic), or CBA/1(third party) mice. Cluster of differentiation 4 receptors positive (CD4+) and CD8+T cells in recipient mice were evaluated. Corneal graft survival was assessed using Kaplan–Meier survival curves.

**Results:**

SEB pre-treatment induced higher levels of hematopoietic chimerism on Days 14, 28 and 56 post-BMT than did CYP or NS pre-treatment. Mean corneal allograft survival was significantly prolonged with group SEB-BMT (20.3±7.6 days) compared to group CYP-BMT (13.0±4.0 days) and NS-BMT (9.0±2.2 days). SEB-BMT mice splenocytes had diminished MLR responses compared to CYP-BMT or NS-BMT mice. CD4+ and CD8+ T cells in peripheral blood and spleens were significantly reduced in group SEB-BMT mice.

**Conclusions:**

BMT after SEB pre-treatment could promote mixed chimerism, which inhibited allogeneic cornea transplant rejection. This should possibly relate to CD4+ and CD8+ T cell deletion and acquiring donor-specific immunosuppression.

## Introduction

Solid organ transplantation is an accepted treatment for end-stage organ failure. Orthotopic allogeneic corneal grafts are among the most successful of solid organ transplants [1]. However, a significant percentage of these grafts are rejected at least once due largely to the unique biology involved as compared to transplanting solid vascularised organs for which systemic immunosuppression is used [2]. When allogeneic corneas are placed in mouse eyes with neovascularized corneas, a situation resembling high-risk eyes in clinical ophthalmology, the incidence and vigor of graft rejection are increased, indicating compromised immune privilege [3]. Thus, methods are needed to overcome the unique immunological barriers involved with corneal transplantation without long-term systemic immunosuppression, which can often have debilitating and possibly fatal consequences [4]. One approach is to induce donor-specific immune tolerance in a graft recipient.

Mixed chimerism and donor-specific tolerance across major histocompatibility complex (MHC) barriers can be induced by donor bone marrow transplantation (BMT) under short-term immunosuppression [5]. However, if conventional doses of bone marrow are used, recipient conditioning with total body irradiation or cytotoxic drugs is usually required. To decrease the toxicity associated with pre-treatment regimens, various protocols, including anti-lymphocyte serum, chemotherapeutic drugs and monoclonal antibodies, have been used to induce bone marrow macrochimerism, primarily in murine models [6-13].

In previous investigations, we used treatments with the superantigen *Staphylococcal* enterotoxin B (SEB) to suppress immune rejection during corneal transplantation [14-17].

SEB is a bacteria-derived superantigen that bypasses classical donor MHC class I and II restrictions and interacts directly with both cluster of differentiation 4 receptors positive (CD4^+^) and CD8^+^ T cells. Of note, T cells respond to SEB stimulation with profound cytokine production by both CD4^+^ and CD8^+^ T subpopulations, which results in T-cell deletion and anergy. We recently showed that SEB significantly prolonged the survival time of allografts in high risk rat corneal allo-transplantation, possibly due to T cell deletion and the acquisition of non-specific tolerance [14]. This suggested that non-myeloablative pre-treatment with SEB could provide a certain period of immunosuppression and raised the question of if this period was sufficient for donor bone marrow to establish a chimera during a period of T cell depletion and anergy.

In this study, we investigated if short-term immunosuppression and anergy induced by BMT after SEB pre-treatment could improve the rate of chimeric establishment and corneal allograft survival in a murine model. As a positive control, we used cyclophosphamide (CYP), a commonly used chemotherapeutic drug that can induce allograft tolerance [18-20].

## Methods

### Mice

Six to 8 week-old female BALB/c (H-2d) and C57BL/6 (H-2b) mice were purchased from The Capital Medical University (Beijing, China). BALB/c mice were used as both bone marrow and cornea donors and C57BL/6 mice were recipients. They were maintained in a specific pathogen-free facility at the vivarium of the Capital Medical University and treated according to the criteria outlined in the National Guidelines for the Care and Use of Laboratory Animals.

### Pre-treatment and bone marrow transplantation

To prepare bone marrow cells (BMCs) for transplantation, unseparated BMCs were harvested from the tibias and femurs of fully MHC-II and minor histocompatibility antigen-mismatched female BALB/c donors [21]. Cells in suspension were counted using trypan blue exclusion (Life Technologies, Inc.). After centrifugation at 1,200× g at 4 °C for 10 min, the BMC pellet was resuspended in 2 ml PBS and adjusted to 4×10^8^ cells/ml. Age-matched female C57BL/6 mice were injected with a total of 25×10^6^ cells/mouse of unseparated BMCs (Day 0) via a caudal vein using a 26-G needle (BD, Inc., Franklin Lakes, NJ).

**Figure 1:**
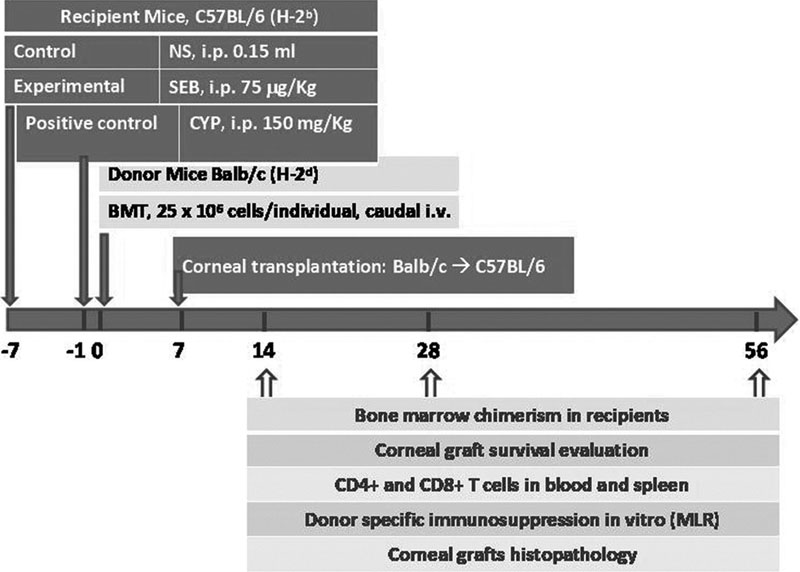
Schematic presentation of the study design. As described in the Methods, C57BL/6 mice received BMCs on Day 0 from BALB/c donors. Prior to BMT, 3 groups of C57BL/6 mice (20 mice/group) were pre-treated with either SEB (Day −7), CYP (Day −1) or Normal Saline (NS; Day −7). Another 3 groups received either SEB (Day −7), CYP (Day −1) or NS (Day -7) without BMT were used as control. At 7 days post-BMT, each C57BL/6 mouse received a corneal transplant from a BALB/c donor. Bone marrow chimerism in recipients was evaluated on Days 14, 28, and 56. Other tests were done post-BMT as described in the Results.

As outlined in Figure 1, three different non-myeloablative pre-treatments combined with or without BMT were used for mice that were to receive corneal transplants. Recipient C57BL/6 mice were divided into 6 groups for different pre-treatments (20 mice/group): SEB treated; CYP treated (positive control group); and normal saline (NS) treated (untreated control group). SEB (Department of Immunology, General Hospital of PLA, Beijing, China) was dissolved in saline and intraperitoneally injected at a single dose of 75 μg/kg at 7 days before BMT (Day −7). CYP (Sigma-Aldrich, St. Louis, MO) was dissolved in saline and intraperitoneally injected at 150 mg/kg at 1 day before BMT (Day −1). NS was intraperitoneally injected at a single dose using a volume of 0.15 ml, similar to the volume used for SEB, at 7 days before BMT (Day −7).

On Day 0 in Figure 1, three groups C57BL/6 mice after different pre-treatment received BMT and the other three groups did not received BMT, then corneal transplantations were performed on Day 7. All recipients were evaluated by flow cytometry to determine if donor-derived hematopoietic chimerisms were established (see below).

### Corneal transplantation

Corneal transplantation used a BALB/c corneal graft as donor and a C57BL/6 mice as recipient. Donor BALB/c central corneas were marked with a 2.0 mm diameter microcurette, excised with a vannas scissors and placed in Optisol-GS (OGS; Bausch & Lomb Surgical, Irvine, CA). Recipients were anesthetized by intraperitoneally injection of ketamine and xylazine, and a 2.0 mm diameter piece in the central cornea was excised from the right eye to prepare the graft bed. The donor cornea was placed in the recipient bed and secured with interrupted 11–0 nylon sutures (Alcon, Fort Worth, TX). After applying an antibiotic ointment, the eye lids were closed for 3 days. Sutures were removed 7 days later. The degrees of graft opacity were assessed using slit lamp biomicroscopy [22]. Briefly, graft opacity was scored using an opacity scale from 0 to 5+. Zero (0) represents a clear graft; 1+=minimal superficial (non-stromal) opacity; 2+=minimal deep stromal opacity; 3+=moderate stromal opacity; 4+=intense stromal opacity; 5+=maximum stromal opacity. A score >2+ indicated a cornea in rejection phase [14]. The mean corneal transplant survival time for each group was also determined.

### Characterization of bone marrow chimerism by flow cytometry

Engraftment of donor BMCs was evaluated by flow cytometry to determine the percentage of donor-derived leukocytes in the peripheral blood of recipients on days 14, 28, and 56 after BMT [20]. Briefly, 500 μl of peripheral blood was collected in a heparinized tube, RBCs were lysed with erythrocyte lysis buffer and the cell suspension was washed twice. Before specific monoclonal antibodies were added the cell suspension was incubated with the Purified Rat anti-mouse CD16/CD32 (Mouse BD Fc Block, BD PharMingen, San Diego, CA) for 15 min. Donor-derived cells were differentiated using a phycoerythrin (PE)-conjugated anti-MHC class II H2d antibody. The chimeric cell's lineage was identified by double staining using PE-labeled anti-MHC class II H2d in combination with peridinin chlorophyll protein (PerCp)-conjugated anti-CD4 mAb, fluoresceine isothiocyanate (FITC)-conjugated anti-CD8a mAb and corresponding isotype controls. All antibodies were from BD PharMingen (San Diego, CA). Fluorescent signals were analyzed with a Becton Dickinson FACScalibur (San Jose, CA). Data analysis used either Cell Quest (Becton Dickinson) software; 10×10^6^ events were acquired for each analysis.

### Determination of chimerism

The percentage of peripheral blood lymphocytes that expressed MHC class II in chimeric mice was determined by labeling with antibodies to H2-b (C57BL/6, host) and H2-d (BALB/c, donor). Because <100% of cells expresses MHC class II antigens, the relative percentage of donor-origin cells in chimeric recipients was calculated by: proportion=[% donor cells/(% donor cells+% host cells)] × 100. Based on isotype control staining, we defined chimeric recipients as those mice in which the percentage of donor-origin cells in the peripheral blood was *>*0.10% on days 14, 28, and 56.

### Mixed lymphocyte reactions (MLR)

MLR assays for BMT recipients were done as described previously [23]. Responder cells were prepared from bone marrow recipients on day 28 (day 21 after corneal transplantation). To detect proliferation toward alloantigens, 5×10^5^ responder cells (splenocytes prepared from C57BL/6 recipients) per well in 200 μl of medium were co-cultured for 4 days with 5×10^5^ irradiated (20 Gy) splenocytes prepared from C57BL/6 (isogeneic), BALB/C (allogeneic), and CBA/1 (third-party) mice as stimulators. Proliferative responses of recipients’ splenocytes were determined with a 3-(4,5-dimethylthiazole-2-yl)-2,5-biphenyl tetrazolium bromide (MTT) assay [18]. All cultures were done in replicates of 5, and mean Optical density(OD±SD) was measured at a wavelength of 570mm by ELISA reader (Thermo MSS; Thermo).

### Corneal allograft histopathological assessments

On day 21 (14 days after corneal transplantation), 2 eye globes from each group of mice were embedded in paraffin, sectioned at 8 μm thickness, and stained with hematoxylin and eosin (H&E). Images were captured under a bright-field microscope (Leica DM 4000B, Leica Microsystems CMS GmbH, Wetzlar, Germany) with a color CCD camera (Leica DFC 300 FX, Leica Microsystems).

### Statistical analysis

Results were given as means±standard deviations (SDs). Unless stated otherwise, groups were compared by ANOVA, with group effects adjusted by Bonferroni’s method. Graft survival was evaluated by generating Kaplan–Meier survival curves, with group comparisons made by a log-rank test. p-values *<*0.05 were considered statistically significant. Statistical analysis used SPSS 15.0 (SPSS, Inc. Chicago, IL).

## Results

### Allogeneic chimerism in bone marrow recipients

As described in the Methods and outlined in Figure 1, before corneal transplantation, C57BL/6 (H-2b) recipient mice received BMT using BMCs from BALB/C (H-2d) donor mice. The percentages of chimerism in recipients were determined by evaluating their peripheral blood cells for donor MHC class H-2d.

**Figure 2:**
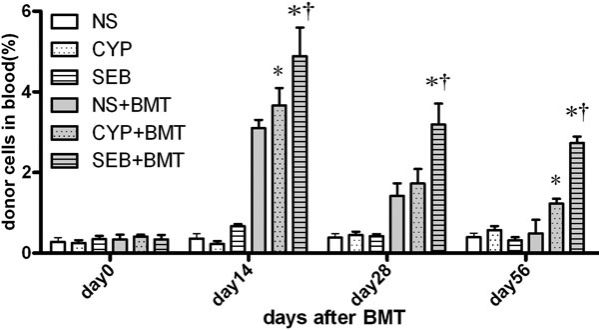
Allogeneic chimerism in bone marrow recipients. Peripheral blood samples from BMT recipients were collected on Day 0, 14, 28, and 56 after BMT. Flow cytometric analysis of blood chimerism was determined using a monoclonal antibody against donor MHC class II H-2d. * p<0.05, significantly different from NS-BMT after Bonferroni’s adjustment at each time point. † p<0.05, significantly different from CYB-BMT after Bonferroni’s adjustment at each time point.

Figure 2 shows the establishment of donor chimerisms in recipient mice on Days 0, 14, 28, and 56 after BMT. The chimerism percentages were significantly higher in the SEB-BMT and CYP-BMT groups than in the NS-BMT group on Days 14 and 28. The chimerism percentages tended to decline after Day 14, although the percentages in the SEB-BMT group remained significantly higher than in the CYP-BMT and NS-BMT groups by Day 56. Thus, SEB pre-treatment prolonged allogeneic chimerism in recipients after BMT to a greater extent than the other pre-treatments.

### CD4+and CD8+T cells in the peripheral blood and spleen of bone marrow recipients

Table 1 shows the percentages of CD4+ and CD8+ T cells in peripheral blood or spleens from the 6 groups of recipient mice at different times after BMT. On days 14 and 56, the percentages of peripheral CD4+ T cells were significantly decreased in the SEB- BMT group compared to the CYP-BMT, NS-BMT, and SEB groups. On day 28 and day 56, the percentages of peripheral CD8+ T cells were decreased in SEB-BMT group compared to the CYP-BMT, NS-BMT, and SEB groups.

**Table 1:** Percentages of T cell subsets in the blood and spleens of recipient mice.

		**Peripheral blood**		**Spleen**	
**Group**	**Days**	**CD4+**	**CD8+**	**CD4+**	**CD8+**
NS	14	30.2±1.9	16.9±0.9	26.8±1.0	11.1±0.8
	28	28.9 ±3.3	16.7±1.3	25.8 ±1.9	13.0±1.7
	56	27.9±1.9	17.6±1.8	27.3±1.8	12.9±1.8
CYP	14	27.8±3.5	15.8±2.5	25.2±3.0	16.9±1.9
	28	27.2 ±2.1	21.0±2.8	29.7±2.7	18.2±1.8
	56	27.5±2.5	19.1±1.4	32.6±2.4	13.2±1.6
SEB	14	28.5±1.5	22.0±2.1	24.8±2.7	13.3±0.7
	28	25.0 ±1.7	16.1±2.5	29.2±1.1	17.4±1.2
	56	23.4±1.8	18.0±1.2	27.2±1.3	17.4±1.1
NS-BMT	14	28.9±2.9	26.5±1.6	42.6±4.5	15.4±2.8
	28	19.2±2.6	13.7±1.3	34.4±3.5	22.0±1.5
	56	22.8±2.0	16.2±1.9	36.3±2.0	18.4±1.4
CYP-BMT	14	25.0±3.6*	21.6±2.2*	36.1±5.8*	14.1±2.5
	28	17.2±2.1*	12.2±0.6	31.5±6.6	21.2±3.3
	56	20.4±3.4*	16.7±1.1	29.5±3.4*	15.0±1.9*
SEB-BMT	14	19.0±2.5*†¢	19.0±1.1*†¢	27.9±8.4*¢	14.4±1.5
	28	17.2±1.5 *¢	13.2±1.2¢	26.6±4.0 *†¢	20.5±3.4¢
	56	19.6±0.9*†¢	15.3±3.1¢	27.7±2.3*†¢	11.6±0.3*†¢

Note: NS, normal saline; CYP, cyclophosphamide; SEB, Staphylococcal Enterotoxin B; BMT, bone marrow transplantation. * p<0.05, significantly different from NS-BMT group after Bonferroni’s adjustment at each time point; † p<0.05, significantly different from CYP-BMT group after Bonferroni’s adjustment at each time point; ¢ p<0.05, significantly different from SEB group after Bonferroni’s adjustment at each time point.

On day 14, the percentages of splenic CD4+ T cells were lower in the SEB-BMT group than in the NS-BMT and SEB groups. On day 28 and day 56, the percentage of splenic CD4+ T cells was significantly lower in the SEB-BMT group than in the CYP-BMT, NS-BMT, or SEB group. Also on day 56, the percentages of splenic CD8+ T cells were lower in the SEB-BMT than in the CYP-BMT, NS-BMT, and SEB groups.

### Donor-specific immunosuppression assessed by MLR

**Figure 3:**
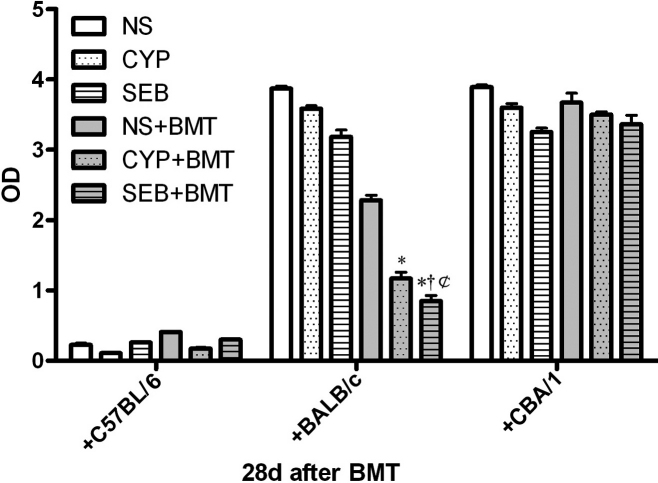
Mixed lymphocyte reactions (MLR) of recipients after BMT. Responder splenocytes were prepared from recipients at Day 28 after BMT. These were co-cultured with irradiated host (C57BL/6), donor (BALB/c), and third-party (CBA/1) splenocytes for MLR assays. Results are the means ± SDs for 5 samples for each group. * p<0.05, significantly different from NS-BMT after Bonferroni’s adjustment for each co-culture. † p<0.05, significantly different from CYB-BMT after Bonferroni’s adjustment for each co-culture. ¢ p<0.05, significantly different from SEB group after Bonferroni’s adjustment for each co-culture.

Mixed allogeneic mice chimeras were assessed for donor-specific immunosuppression in vitro using MLR. Responder cells were prepared from the 6 pre-treatment groups of C57BL/6 recipient mice on day 28 . Stimulator cells were prepared from C57BL/6 host mice (isogeneic), BALB/c donor mice (allogeneic) or CBA/1mice (third-party). Figure 3 shows the proliferative responses of responder cells from recipient mice to the different stimulator cells.

Using cells from C57BL/6 mice as stimulators, there were slight differences in proliferation among the responder cells from the 6 pre-treatment groups; however, the overall proliferative responses were minimal. In contrast, responder cells from all 6 groups exhibited significant proliferation when stimulated by cells from third-party CBA/1 mice but there were no significant difference among all groups. Using stimulator cells from BALB/c donor mice, the proliferative responses were significantly different among the responder cells from the 6 pre-treatment groups; in particular, responder cells from the SEB-BMT group exhibited the lowest proliferative responses to BALB/c donor cells. Thus, SEB pre-treatment along with donor BMT induced a higher degree of donor-specific immunosuppression in recipient mice.

### Corneal allograft survival

As outlined in Figure 1, after the different pre-treatment regimens, C57BL/6 recipient mice received corneal transplants from BALB/c donors at Day 7 after BMT. Allograft survival was assessed up to Day 56. The mean allograft survival times (in days) for the 6 groups of recipient mice were: 7.4±1.5 for NS mice; 9.3±1.7 for CYP mice; 11.2±2.1 for SEB mice; 9.0±2.2 for NS-BMT mice; 13.0±4.0 for CYP-BMT mice, and 20.3±7.6 for SEB-BMT mice. These survival times were significantly different among the 6 groups (p<0.05).

**Figure 4:**
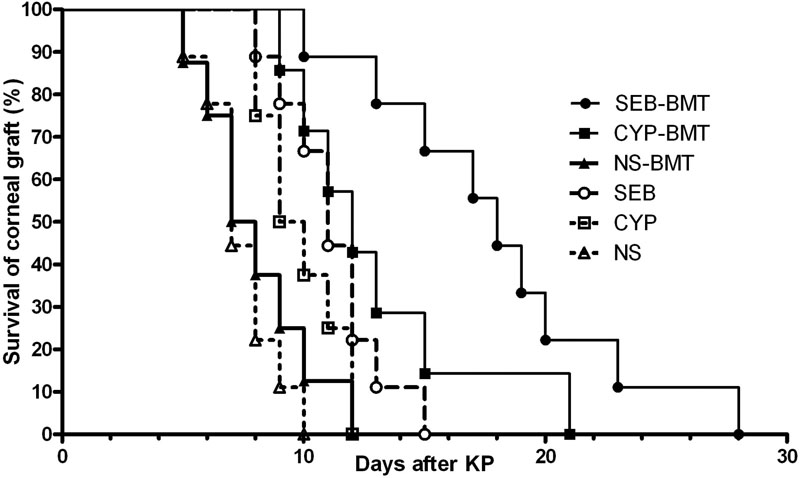
Corneal allograft survival. Kaplan–Meier survival curves for corneal allografts transplanted into recipients after pre-treatment along with BMT. PK=penetrating keratoplasty.

**Figure 5:**
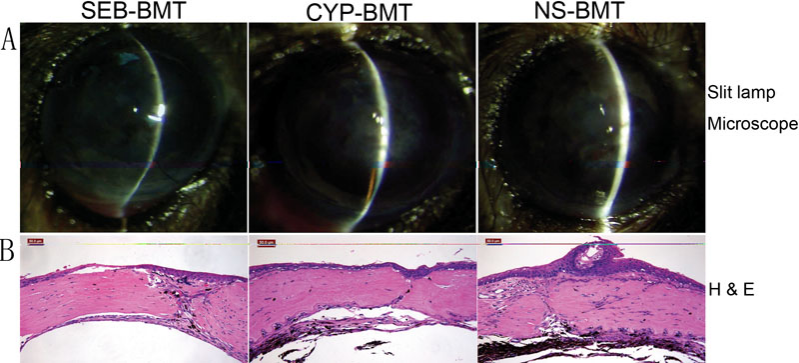
Clinical examination by slit lamp microscopy and histopathology of corneal allografts. **A**: Representative images of the corneal allograft in each experimental group. **B**: Histopathological evaluation for corneal sections using H&E staining (40×). Graft rejection was observed in the corneal necropsy specimens of the BMT recipients for the SEB-BMT, CYP-BMT, and NS-BMT groups by Day 21 after BMT (Day 14 after corneal transplant). Lymphocyte infiltration and neovascularization were significantly decreased in the SEB-BMT group compared to the CYP-BMT and NS-BMT groups.

To better illustrate these differences, Figure 4 shows Kaplan–Meier survival curves for allograft survival. Corneal allograft survival was the longest for mice pre-treated with SEB and that received BMT from allograft donor mice. After surgery, mice were given clinical examinations by slit lamp microscopy every few days for 56 days for allograft survival using a previously described scoring system to evaluate corneal grafts infiltration (opacity) [14]. Representative images of corneal allografts are shown in Figure 5A.

### Corneal allograft histopathological assessments

As described in the methods, on day 21 (14 days after corneal keratoplasty), 2 eyes from each group of mice were embedded in paraffin, sectioned at 8 μm thickness, followed by H&E staining. These sections were used for histopathological assessments. Figure 5B shows representative H&E stained sections for the 3 pre-treatment BMT groups. Different degree of tissue damage was observed in the corneal allograft autopsy specimens of the bone marrow recipients for the SEB-BMT, CYP-BMT, and NS-BMT groups. However, lymphocyte infiltration and neovascularization were significantly reduced in the SEB-BMT group compared to the CYP-BMT and the NS-BMT groups (Figure 5B).

## Discussion

In this study, we addressed the question of whether a single pre-treatment dose of the bacterial superantigen SEB before BMT would provide a “window of opportunity” in recipient mice in terms of inducing donor-specific immunosuppression that would be sufficient to enhance the establishment of chimerism and promote corneal allograft survival. Although the detailed mechanisms will require additional investigations, our results showed that, as compared to pre-treatment with either CYP or NS, SEB pre-treatment did enhance the establishment of chimerism in BMT recipients and prolonged the period of corneal allograft survival in these recipient mice. These effects appeared to be due, at least in part, to the effects of SEB to reduce reactive clones of CD4+ and/or CD8+ T cells in recipient mice.

Induction of donor-specific tolerance can promote allografts to be accepted across MHC barriers without requiring chronic immunosuppressive therapy. Among numerous attempts to induce organ allograft tolerance, one commonly accepted method involves the infusion of donor-derived cells, particularly BMCs, associated with various immunologic manipulations [24]. It has been demonstrated that robust and lifelong donor-specific tolerance could be achieved by the induction of chimerism in various animal models. Recipient irradiation or other myeloablative therapy is usually necessary to achieve engraftment success.

In initial attempts with rodents, mixed allogeneic chimerism was established using lethal irradiation and reconstitution of the recipient with a mixture of T-cell depleted host and donor BMCs [25]. Subsequently, to eliminate the need for lethal irradiation, ablation used different regimens of irradiation with or without cytotoxic drugs and cytotoxic antibodies [26,27]. Thymic irradiation or ablation with cytotoxic antibodies was also used in some cases to create ‘‘space’’ in the recipient thymus, so that lasting central tolerance could be achieved [28-30]. However, the mechanisms underlying these phenomena are not fully understood, and they still require administering potentially harmful pre-conditioning regimens.

Recent reports have shown that SEB superantigens may shape the T cell repertoire by the deletion or inactivation (anergy) of reactive clones. Cellular unresponsiveness and concomitant down-regulation of T cell receptors (TCR) and/or co-receptors have been observed in some experimental models of tolerance in vitro [31] and in vivo [32], suggesting that receptor down-modulation may be of functional significance for cellular inactivation. This may involve down-regulation of TCRs and/or CD4 or CD8 co-receptors, as well as other intracellular mechanisms, and would condition the capacity of T cells to be reactivated.

Our previous studies with rats showed that· SEB could prolong graft survival time by inducing T cell deletion and non-specific tolerance in high risk corneal transplantation [14-17]. Because the mechanisms involved are different between SEB pre-treatment and the induction of donor-derived hematopoietic chimerism, the question arose of whether we could combine these two approaches to achieve enhanced immune tolerance? Our current experiments demonstrated that the chimerism rate of donor derived BMC engraftment was increased in the SEB pre-treated group, which appeared to be greater than that with CYP pre-treatment. This indicated that SEB pre-treatment could promote BMC chimerism formation.

In addition, the SEB pre-treated group with BMC engraftment also exhibited a longer allograft survival time compared to the NS pre-treated BMT group or SEB pre-treated without BMT group. This indicated that the combination of SEB pre-treatment and BMC engraftment could induce donor-derived specific immunosuppression and promote corneal allograft survival. However, our animal model was normal penetrating keratoplasty, more works should be done to verify if this SEB pre-treated with BMC engraftment could inhibited high-risk corneal grafts rejection.

Previous reports showed that either SEB treatment or BMC engraftment could manipulate T cell responses, particularly for the CD4^+^ and CD8^+^ populations. We also observed that the CD4^+^ and CD8^+^ populations were affected by the BMT after pre-treatments used. The percentage of CD4+ or CD8+ T cells in peripheral blood in SEB-BMT group was the lowest in all groups. Their percentages of splenic CD4^+^ and CD8^+^ T lymphocytes were also reduced significantly. Thus, SEB pre-treatment could reduce the populations of CD4+ and CD8+ T cells both in the peripheral circulation and spleens of recipient mice after BMT, which would be related with corneal grafts survival time prolongation.

Our results with MLR also showed reduced T lymphocyte reactivity resulting from BMT after SEB pre-treatment. In addition to reduced lymphocyte numbers by SEB pre-treatment, the lymphocytes proliferative capacity was also reduced when stimulated by donor antigens. We called this condition immunosuppression but not immune tolerance because the recipient mice corneal grafts rejection happened at last. We also found that lymphocyte infiltration into the corneal grafts was significantly decreased when recipient treated with BMT after SEB pre-treatment. This results implied that SEB-BMT should have induced donor-specific immunosuppression in mice receiving corneal transplants.

In summary, our results showed that a single-dose of SEB pre-treatment could efficiently enhance the establishment of chimerism by donor bone marrow engraftment, which could induce donor-specific immunosuppression and prolong corneal graft survival. This may have been related to CD4^+^ and CD8^+^ T cell deletion. The mechanisms of how SEB combined with BMC transplantation affects cellular immune responses need to be better understood.
